# Psycho-Emotional and Behavioral Problems in Children With Growth Hormone Deficiency

**DOI:** 10.3389/fped.2021.707648

**Published:** 2021-09-23

**Authors:** Mykola Aryayev, Liudmyla Senkivska, John B. Lowe

**Affiliations:** ^1^Department of Paediatrics, Odessa National Medical University, Odessa, Ukraine; ^2^School of Health and Behavioural Sciences, University of the Sunshine Coast, Maroochydore, QLD, Australia

**Keywords:** growth hormone deficiency, children, self-esteem, psycho-social problems, Strengths and Difficulties Questionnaire

## Abstract

**Objective:** To identify psychosocial problems and self-esteem in children with growth hormone deficiency (GHD) and define the role of some clinical and sociodemographic determinants in the conceptualization of internalizing and externalizing problems as criteria for psychosocial functioning.

**Materials and Methods:** A GHD sample (46 prepubescent children) was selected and compared to a matched control group (80 healthy children). Psychosocial functioning in children with GHD was investigated using Goodman's “Strengths and Difficulties Questionnaire (SDQ).” The study of children's self-esteem was carried out by the Dembo–Rubinstein method.

**Results:** This study reveals that the GHD sample has more internalizing problems and lower self-esteem. Higher score and frequency of assessment in the abnormal score for “total difficulties,” “emotional problem,” and “peer problem” were found in children with GHD. The SDQ score and the frequency of assessment in the abnormal score for all SDQ scales in children with more pronounced growth deficit (height SDS < −3) did not exceed the same indicators in children with less growth retardation (−3 < height SDS < −2). A comparison of psychosocial features in children with isolated growth hormone deficiency and multiple pituitary hormones deficiency did not reveal differences in SDQ score and the frequency of assessment in the abnormal score for all SDQ scales. It was found that children with GHD have a reduced level of assertions, low self-esteem, and a weak discrepancy between the level of assertions and self-esteem. Some sociodemographic determinants (male gender, age < 9 years, and low family income) and clinical determinants (low compliance and suboptimal growth response after 1 year of rGHh therapy) have an impact on the overall assessment of psychological problems in children with GHD. The internalizing difficulties are associated with certain clinical determinants (growth status and treatment status) and sociodemographic determinants (female gender, age < 9 years).

**Conclusions:** The identification of low self-esteem and the high SDQ score for scales “total difficulties,” “emotional problems,” and “peer problems” indicates psychosocial maladjustment and conceptualization of internalizing problems in children with GHD.

## Introduction

The study of the psychosocial problems and self-esteem in children with growth hormone deficiency (GHD) in the context of comprehensive diagnosis and treatment (1) is an important but understudied area of research. A promising prospect for improving management of GHD is the early identification of children at risk for psychosocial problems and the timely provision of appropriate psychotherapeutic supportive and tailored care. To date, studies conducted in this context are few, and the results have not been unambiguously interpreted (2, 3). The Strengths and Difficulties Questionnaire (SDQ) provides balanced coverage of children and young people's behaviors, emotions, and relationships (4, 5). The SDQ covers hyperactivity/inattention, conduct problems, and emotional and peer problems that are not well-understood in children with GHD. There is theoretical and preliminary empirical support for combining the SDQ's hypothesized emotional and peer scales into an “internalizing” scale and the hypothesized behavioral and hyperactivity scales into an “externalizing” scale (6). There may be benefits in assessing psychosocial functioning using the SDQ method in conjunction with the determination of self-esteem among children with GHD, but to the best of our knowledge, no such complex studies have been conducted before.

### Objective

To identify psychosocial problems and self-esteem in children with GHD and define the role of some clinical and sociodemographic determinants in the conceptualization of internalizing and externalizing problems, as criteria for psychosocial functioning.

## Materials and Methods

An open comparative clinical study was performed at the Department of Endocrinology of the Odessa Regional Children's Clinical Hospital (Odessa, Ukraine) in 2012–2020 (with the permanent inclusion of new patients). Among 92 children with GHD (23 girls and 69 boys), all prepubescent children were selected from the full group for psychological studies (*n* = 46; 12 girls, 34 boys). The age range was 8.0–12.1 years (M = 9.8; SD = 3.1). The diagnosis of GHD was based on an integrated assessment of clinical signs, auxological measurements, growth deficit, annual growth rate, bone age, MRI of the skull, and growth hormone release <10 ng/ml in provocative testing using insulin and clonidine. Basic therapy was performed with recombinant human growth hormone (rHGh) at an average dose of 0.033 mg/kg/day. The control group included 80 healthy children aged 8.1–12.0 years (M = 9.6; SD = 2.7). These children were examined on the “healthy child's day” at the outpatient Department of the Odessa Regional Children's Clinical Hospital. The average height of this group was normal. There were none with other conditions for which rhGH is an appropriate therapy in the study design. The study was conducted in accordance with the principles of the Helsinki Declaration and the standards of Good Clinical Practice. The informed consent of parents and children has been obtained.

Psychosocial functioning was assessed using the “Strengths and Difficulties Questionnaire” (SDQ), developed by Goodman et al. (4). The Questionnaire contained 20 items distributed on four scales: (1) emotional problems, (2) behavioral problems, (3) hyperactivity/inattention, and (4) peer problems. The answer to each question was given points according to the scoring system: 0 = incorrect, 1 = partially true, and 2 = true. The scores for hyperactivity/inattention, emotional problems, behavioral problems, and peer problems can be summed to generate a total difficulties score in the range of 0–40 points. In addition, it is possible to calculate scores for internalizing by summing scale scores of emotional problems and peer problems (in the range of 0–20 points) and externalizing by summing scale scores of behavioral problems and hyperactivity/inattention (in the range 0–20 points). The results were marked as normal, borderline, or abnormal. Normal scores on scales “total difficulties,” “emotional problems,” “behavioral problems,” “hyperactivity/inattention,” and “peer problems” were 0–14, 0–4, 0–3, 0–5, and 0–3 points, respectively. Borderline scores were defined as 15–16, 5, 4, 6, and 4 points, respectively, and abnormal scores 17–40, 6–10, 5–10, 7–10, and 5–10 points, respectively. The questionnaire took from 5 to 10 min to complete. The SDQ scores were analyzed as continuous variables, with higher scores indicating more difficulties.

Self-esteem of children with GHD was studied by the Dembo-Rubinstein method (7). The children filled out a form with seven vertical lines (scales), 100 mm in size. The lower point of the scale indicates the lowest score, and the upper indicates the highest. The child marks with a dash (–) his position (development of each quality) at a given time. The self-esteem value is determined from the lower point of the scale (0) to the dash (–) in mm and is translatEd into points (100 mm = 100 points). The child indicatEs with a sign (x) on the same line the level of claims, that is, what in his opinion should be the appropriate quality. The level of claims for this quality is estimated from (0) to the sign (x) in mm and translated into points.

Statistical processing of the results using the criterion “xi-square χ^2^” and by estimating the differences between the mean values of two independent variation series by the value of “*p*” was performed. SDQ scores were used as predictor variables. The association between clinical and sociodemographic determinants and psychosocial problems was studied using the odds ratio (OR). A singular effect of each variable was analyzed (8).

## Results of the Research

A peculiarity of using the A. Goodman's questionnaire in this study was the assessment of psychosocial problems both within the generally accepted four-factor structure of SDQ (9) and on the basis of bifactor SDQ model of externalizing and internalizing problems (4, 10, 11). The last model allows to assess the conceptualization of the externalizing and internalizing difficulties in children with GHD and the impact of some clinical and sociodemographic factors.

SDQ testing revealed an increase in the score for the overall assessment of psychosocial problems (“total difficulties”) and the scores for “emotional problems” and “peer problems” in children with GHD. Higher scores on the SDQ scales mean a greater severity of relevant problems and therefore have a screening value (Table 1). The results provide an opportunity to identify the problems of internalizing. Externalizing problems score had no statistical differences between the main and control groups.

**Table 1:** SDQ score for psychosocial problems in children with GHD and in the control group.

**SDQ score**	**Control group** (***n*** **= 80) M ± SD**	**Main group** (***n*** **= 46) M ± SD**	***P***
Total difficulties score	10.2 ± 5.6	13.0 ± 9.1	0.03
Hyperactivity/inattention score	3.7 ± 1.9	4.3 ± 3.2	0.19
Behavioral problems score	2.0 ± 1.5	2.1 ± 1.3	0.70
Emotional problems score	2.4 ± 2.0	3.5 ± 3.1	0.02
Peer problems score	2.1 ± 1.6	3.1 ± 3.0	0.01
Internalizing problems score	4.5 ± 1.7	6.6 ± 5.3	0.001
Externalizing problems score	5.7 ± 3.4	6.4 ± 3.3	0.26

The frequency of assessment of psychosocial problems in the normal, borderline, and abnormal scores for the SDQ scales in children with GHD was compared with the results in the control group (Table 2). The frequency of the “total difficulties” in the abnormal score in children with GHD significantly exceeded the studied parameter in the control group, according to the χ^2^ test. The combination of a higher frequency of the “emotional problems” and “peer problems” in the abnormal scores in children with GHD, compared with the control group, identifies the internalizing problems. No significant differences were found between children with isolated growth hormone deficiency and multiple pituitary hormone deficiency in the frequency of assessment for abnormal score in the areas of “total difficulties,” “emotional problems,” “behavioral problems,” “hyperactivity/inattention,” “peers problems.” The overall assessment of psychosocial problems (“total difficulties”) and the frequency of assessment in the abnormal score for all SDQ scales had no statistical differences when comparing children with height SDS < 3 and −3 < SDS < −2.

**Table 2:** The frequency of psychosocial problems in the normal, borderline, and abnormal scores for the SDQ scales (χ^2^ test).

**Scales and scores**	**Main group (***n*** = 46) ***n***; % (CI 95%)**	**Control group (***n*** = 80) ***n***; % (CI 95%)**	**χ^2^**	***P***
**Total difficulties**				
Normal score	31; 67.4 (53.9–80.9)	67; 83.7 (75.6–91.8)	4.45	0.03
Borderline score	5; 10.9 (1.9–19.9)	6; 7.5 (1.7–13.3)	0.42	0.42
Abnormal score	10; 21.7 (9.8–33.6)	7; 8.7 (2.5–14.9)	4.21	0.04
**Hyperactivity/inattention**				
Normal score	33; 71.7 (58.7–84.7)	66; 82.5 (74.2–90.8)	2.00	0.16
Borderline score	8; 17.4 (6.4–28.4)	7; 8.7 (2.5–14.9)	2.10	0.15
Abnormal score	5; 10.9 (1.9–19–9)	7; 8.7 (2.5–14.9)	0.16	0.69
**Behavioral problems**				
Normal score	36; 78.2 (66.3–90.1)	70; 87.5 (80.3–94.7)	1.87	0.17
Borderline score	7; 15.2 (4.8–25.6)	6; 7.5 (1.7–13.3)	1.86	0.17
Abnormal score	3; 6.5 (−0.6–13.6)	4; 5.0 (0.2–9.8)	0.12	0.72
**Emotional problems**				
Normal score	33; 71.7 (58.7–84.7)	69; 86.2 (78.6–93.8)	3.94	0.05
Borderline score	4; 8.7 (0.6–16.8)	5; 6.3 (1.0–11.6)	0.25	0.62
Abnormal score	9; 19.6 (8.1–31.1)	6; 7.5 (1.7–13.3)	4.04	0.04
**Peer problems**				
Normal score	30; 65.2 (51.4–79.0)	68; 85.0 (77.2–92.8)	6.57	0.01
Borderline score	8; 17.4 (6.4–28.4)	7; (8.7; 2.5–14.9)	2.10	0.15
Abnormal score	8; 17.4 (6.4–28.4)	5; (6.3; 1.0–11.6)	3.85	0.05

The influence of some clinical and sociodemographic determinants on the overall assessment of psychosocial problems (“total difficulties”) and conceptualization of internalizing problems in children with GHD were investigated. The rationale for this approach was a significant increase in the overall assessment of psychological problems and internalizing problems scores. Externalizing problems score had no statistical differences between the main and control groups. The study mainly focused on the bifactor SDQ model but not on separate scales. The group of children with the SDQ total difficulties in abnormal/borderline scores is being compared with the group with normal scores. The group of children with the SDQ internalizing problems in abnormal/borderline scores is being compared with the group with normal scores. Age, gender of the child, family income, marital status, and parental education were studied as sociodemographic determinants. Therapeutic status and growth status of patients were considered as potential clinical determinants. The therapeutic status included comorbidity and low compliance to growth hormone therapy according to the Morisky Medication Adherence Scale. Growth status was established by the fact of suboptimal growth response (delta height SDS < 0.5 after 1 year of the replacement therapy).

Some sociodemographic determinants (male gender, age < 9 years, and low family income) have an impact on the overall assessment of psychological problems (SDQ total difficulties score) in children with GHD. A correlation between some clinical determinants (low compliance and suboptimal growth response after 1 year of rhGH therapy) and the SDQ total difficulties score was also found (Table 3).

**Table 3:** The influence of some clinical and sociodemographic determinants on the overall assessment of psychological problems in children with GHD.

**Sociodemographic/clinical determinants**	**OR (CI 95%)**	***P***
Male gender	2.2 (1.4–3.3)	0.001
Age < 9 years	1.6 (1.0–2.5)	0.05
Below average income	1.7 (1.1–2.6)	0.05
Incomplete family	1.5 (0.8–3.2)	0.10
Mother's education above secondary	0.5 (0.1–1.0)	0.24
Father's education above secondary	0.7 (0.3–1.9)	0.18
Comorbidity	1.6 (0.8–4.0)	0.08
Low adherence to GH therapy	1.8 (1.2–2.0)	0.001
Suboptimal growth response	2.0 (1.8–2.3)	0.001

Certain sociodemographic determinants (female, age < 9 years) were associated with internalizing difficulties (SDQ internalizing problems score) (Table 4). No correlation between the problems of internalizing and the marital status of parents, family income, and educational level of parents was found. The treatment status (low adherence to GH therapy) and growth status (suboptimal growth response to rhGH therapy within 12 months) have an impact on internalizing problems. The association between the SDQ internalizing problems score and comorbidity has not been confirmed.

**Table 4:** The influence of some clinical and sociodemographic determinants on the assessment of internalizing problems in children with GHD.

**Sociodemographic/clinical determinants**	**OR (CI 95%)**	***P***
Female	1.8 (1.2–2.8)	0.05
Age <9 years	2.4 (1.5–3.8)	0.01
Below average income	1.3 (0.7–2.1)	0.08
Incomplete family	1.5 (0.7–2.7)	0.06
Mother's education above secondary	0.4 (0.2–0.8)	0.12
Father's education above secondary	0.6 (0.2–1.6)	0.14
Comorbidity	1.2 (0.5–2.5)	0.24
Low adherence to GH therapy	2.2 (1.6–3.6)	0.05
Suboptimal growth response	2.5 (1.3–3.2)	0.05

The results of testing children with GHD in the main group compared with the control group revealed a reduced level of assertions, low self-esteem, and a weak discrepancy between the level of assertions and self-esteem (Table 5).

**Table 5:** Level of self-esteem and assertions in children with GHD.

**Indicator**	**Main group (***n*** = 46) ***n***; % (CI 95%)**	**Control group (***n*** = 80)***n***; % (CI 95%)**	***P***
Low level of assertions (<60 points)	9; 19.6 (8.1 ÷ 31.1)	6; 75 (1.7 ÷ 13.3)	0.04
low self-esteem (<45 points)	8; 17.4 (6.4 ÷ 28.4)	5; 6.2 (0.9 ÷ 11.5)	0.05
Weak discrepancy between the level of assertions and self-esteem (<7 points)	10; 21.7 (9.8 ÷ 33.6)	7; 8.7 (2.5 ÷ 14. 9)	0.04

## Discussion

The nature of the relationships between short stature of children and psycho-emotional problems and behavior remains the subject of scientific debate. Negative social effects of short stature became known; however, the authors of a number of studies consider that short stature as an isolated physical characteristic is not directly related to psychosocial maladjustment (12). On the other hand, a number of studies have established the role of short stature as a cause of chronic psychosocial stress, impaired psychosocial adaptation, cognitive impairment, poor performance, and reduced intellectual abilities (13). Many children with chronic disease suffer from distinct emotional, social, and behavioral problems (14). This study is connected to previous literature and embedded in ongoing discourse. It confirms the medical and social significance of short stature and corresponds to the data of Quitmann et al. (3) and Chaplin et al. (15) on reducing self-esteem in children with GHD and improving psychosocial well-being with optimal growth response to rhGH therapy. The study shows the role of sociodemographic factors rather than height-related aspects in explaining psychosocial outcomes (16). The examination of the Ukrainian GHD sample adds to the literature and supports research and treatment processes.

To our knowledge, this is the first study to examine both psychosocial problems and self-esteem conjointly as indicators of psychosocial adaptation in children with GHD. The results suggest that children with GHD are at greater risk for internalizing problems. The research revealed an association between some clinical/sociodemographic determinants and the psychosocial problems/internalizing problems in children with GHD. The “total difficulties” score and the frequency of assessment of psychosocial problems in the abnormal score for all the SDQ scales in children with more significant growth deficiency (height SDS < −3) did not exceed similar indicators in children with less growth retardation (−3 < SDS < −2). This fact indicates the need for the earliest possible diagnosis of GHD in children in order to timely prescribe replacement therapy and psychological support. The overall assessment of psychosocial problems (“total difficulties”) and the frequency of assessment in the abnormal score for all the SDQ scales in children with isolated growth hormone deficiency and multiple pituitary hormones deficiency were the same. This fact shows the possibility of developing a common psychological support program for patients with various forms of GHD. This study indicates that therapy with optimal growth response helps to restore the psychosocial functioning in children with GHD by reducing the problems of internalizing. Further research is needed on the impact of different types of growth deficits and various forms of GHD on the quality of life and other indicators of psychosocial maladjustment.

The research methods include robust testing with a previously well-described set of instruments. The main focus of this study is based on the SDQ, which was developed primarily as a screening tool for behavioral and emotional problems in children and adolescents, but increasingly being used for assessment of psychosocial problems in the clinical setting (17–19). The bifactor SDQ models of externalizing and internalizing disorders have recently obtained more attention (6, 19). The rationale for combining the subtests to two major categories was provided by the author of SDQ, Goodman et al. (4). The SDQ is widely used, but its validity is not yet given for all contexts and target groups. A study on the validation of the Russian version of the SDQ for instance revealed poor psychometric properties (20). The sufficient internal consistency for all SDQ scales and informativeness of the four-factor structure of the SDQ scale as a rapid method for identifying social, emotional, and behavioral problems have been reconfirmed by Becker et al. (21). The use of Goodman's SDQ has become widespread internationally (22–24), especially among chronically ill patients. The clinical significance of local studies given the diversity of national, ethnic, and regional characteristics of the pediatric population has been demonstrated by a number of studies (25–27). The SDQ seems to be an appropriate instrument to assess psychosocial functioning among children with GHD, which can be used for clinical and research purposes. The use of bifactor SDQ model can be recommended in pediatric practice and social services in Ukraine as a screening tool for the identification of children at risk for psychosocial problems to be able to provide psychological supportive and tailored care at an early stage. Psychological interventions should be considered when clinically indicated.

Although this paper has provided a comprehensive assessment of psychosocial problems, internalizing difficulties, and self-esteem in children with GHD and has made a novel contribution to the literature surrounding the influence of certain clinical and sociodemographic determinants on the psychosocial problems (as captured by SDQ), the study is not without limitations. They include quite a short-term follow-up for children with GHD, no other control groups of children with short stature for whom rhGH is indicated, and age restriction. Prospects for further research are to compare the psychosocial functioning and self-esteem in different groups of short stature patients treated with rhGH and evaluate changes in SDQ score over the years in order to improve the individual psychological support.

## Conclusions

1. The comparison of children with and without GHD in their SDQ scales “total difficulties,” “emotional problems,” and “peer problems” indicates psychosocial maladjustment and conceptualization of internalizing problems in the GHD sample. No externalizing difficulties were revealed in children with GHD.
2. Some clinical determinants (low compliance and suboptimal growth response after 1 year of rhGH therapy) and sociodemographic determinants (male gender, age < 9 years, and low family income) have an impact on the overall assessment of psychological problem in children with GHD that can be taken into account in individual psychological support programs.
3. Children with GHD, whether isolated or part of a multihormonal deficit, have more internalizing problems than a control group. Certain sociodemographic data like female gender and age < 9 years are connected to the internalizing problems in children with GHD. The conceptualization of internalizing problems is also associated with the influence of certain clinical determinants like low adherence and suboptimal growth response to 12 months of rhGH therapy.
4. GHD sample has a reduced level of assertions, self-esteem, and a weak discrepancy between the level of assertions and self-esteem. Early identification of psychosocial problems and low self-esteem can be a part of the routine management and monitoring of therapy for children with GHD.

## Data Availability Statement

The raw data supporting the conclusions of this article will be made available by the authors, without undue reservation.

## Ethics Statement

The studies involving human participants were reviewed and approved by Ethics committee of the Odessa Regional Children's Clinical Hospital (Odessa, Ukraine). Written informed consent to participate in this study was provided by the participants' legal guardian/next of kin.

## Author Contributions

MA contributed to conception and design of the study. LS organized the database and wrote the first draft of the manuscript. JL performed the statistical analysis. All authors contributed to manuscript revision, read, and approved the submitted version.

## Conflict of Interest

The authors declare that the research was conducted in the absence of any commercial or financial relationships that could be construed as a potential conflict of interest.

## Publisher's Note

All claims expressed in this article are solely those of the authors and do not necessarily represent those of their affiliated organizations, or those of the publisher, the editors and the reviewers. Any product that may be evaluated in this article, or claim that may be made by its manufacturer, is not guaranteed or endorsed by the publisher.
